# Smooth muscle-like Ca^2+^-regulation of actin–myosin interaction in adult jellyfish striated muscle

**DOI:** 10.1038/s41598-018-24817-x

**Published:** 2018-05-17

**Authors:** Hiroyuki Tanaka, Shiori Ishimaru, Yasuhiro Nagatsuka, Keisuke Ohashi

**Affiliations:** Laboratory of Marine Biotechnology and Microbiology, Graduate School of Fisheries Sciences, Hokkaido University, Hakodate, Japan

## Abstract

Cnidaria is an animal phylum, whose members probably have the most ancestral musculature. We prepared and characterized, for the first time to our knowledge, native actomyosin from the striated myoepithelium of the adult moon jelly *Aurelia* sp. The actomyosin contained myosin, paramyosin-like protein, Ser/Thr-kinase, actin, and two isoforms of tropomyosin, but not troponin, which is known to activate contraction dependent on intracellular Ca^2+^ signaling in almost all striated muscles of bilaterians. Notably, the myosin comprised striated muscle-type heavy chain and smooth muscle-type regulatory light chains. In the presence of Ca^2+^, the Mg-ATPase activity of actomyosin was stimulated and Ser21 of the regulatory light chain was concomitantly phosphorylated by the addition of calmodulin and myosin light chain kinase prepared from chicken smooth muscle. Collectively, these results suggest that, similar to smooth muscle, the contraction of jellyfish striated muscle is regulated by Ca^2+^-dependent phosphorylation of the myosin light chain.

## Introduction

The contraction and relaxation of musculature are regulated by cytosolic Ca^2+^ concentrations. The Ca^2+^ ion binds to a regulatory protein and initiates sliding of actin and myosin filaments past each other, a process that is coupled to ATP hydrolysis on the myosin molecule. The regulatory protein is divergent among animal phyla and among muscle types. In the many striated muscles of both vertebrates and invertebrates, a troponin–tropomyosin complex on the actin filament binds Ca^2+^ and exposes the myosin-binding site on actin, resulting in the interaction of actin and myosin^1,2^. In smooth muscles, by contrast, Ca^2+^ binds to cytosolic calmodulin (CaM), and the Ca^2+^/CaM complex activates myosin light chain kinase (MLCK)^3,4^. This kinase phosphorylates the regulatory light chain of myosin, resulting in the interaction of actin and myosin^3,4^. Furthermore, in molluscan muscles, Ca^2+^ directly binds to the essential light chain of myosin and activates the contraction^5,6^. In many cases, these different mechanisms of regulation may coexist in the same muscle^2,7^.

The phylum Cnidaria is thought to have arisen 600 million years ago and contains some of the earliest animals to have acquired musculature and a nervous system^8^. Among the Cnidaria, jellyfish has a life cycle involving three morphologically different major stages; namely, planula, polyp, and medusa. The planula migrates by ciliary movement, although in some Hydrozoa, the swimming direction is controlled by a muscle contraction that allows body bending^9^. The polyp contains smooth but not striated muscles in its tentacles, oral disc, and body column, whereas the medusa has both smooth and striated muscles^10^. In early spring, the sessile polyp of the moon jelly *Aurelia* shows transverse segmentation, termed strobilation, which gives rise to many free-swimming larvae called ephyrae. During strobilation, the ephyrae develop both radial and circular striated muscles for swimming^11,12^. -Each ephyra grows into a mature medusa, which has smooth muscles in its tentacles, oral arms, and the marginal region of the subumbrella (underside of the umbrella), as well as striated myofibrils that are arranged concentrically and form a muscular sheet in the subumbrella, the rhythmic contraction of which generates pulsation for swimming^10,13,14^. The striated myofibrils structurally resemble those of vertebrate skeletal muscles; that is, the sarcomere, which comprises interdigitating thick and thin filaments sandwiched between Z-discs, is longitudinally repeated^11,15–17^. However, genome and transcriptome mining has revealed that jellyfish lacks troponin^18^, a protein that has been found in almost all striated muscles of other phyla^2^. In addition, connectin (titin), a large protein that connects the Z-disc and thick filament to maintain the structure of sarcomeres^19^, is missing. On the basis of these observations, the structural similarity between the striated muscles of jellyfish and higher animals is considered to have resulted from convergent evolution^18^.

The absence of troponin indicates that there is likely to be another machinery for the Ca^2+^-dependent regulation of contraction in jellyfish striated muscle. It is important to elucidate the ancestral regulatory mechanisms of cnidarian muscle contraction in order to understand the evolution of muscles in the animal kingdom. To date, however, there have been no reports about the biochemical properties of muscular proteins from jellyfish, probably because both the large amount of gelatinous components and the protease released from the animal’s gastric cavity into its whole body interfere with purification of these proteins. In this study, we prepared and characterized for the first time the native actomyosin from jellyfish striated muscle to investigate the Ca^2+^-dependent regulation of muscular contraction in this species.

## Results and Discussion

### Morphological features of moon jelly striated muscle

**Figure 1:**
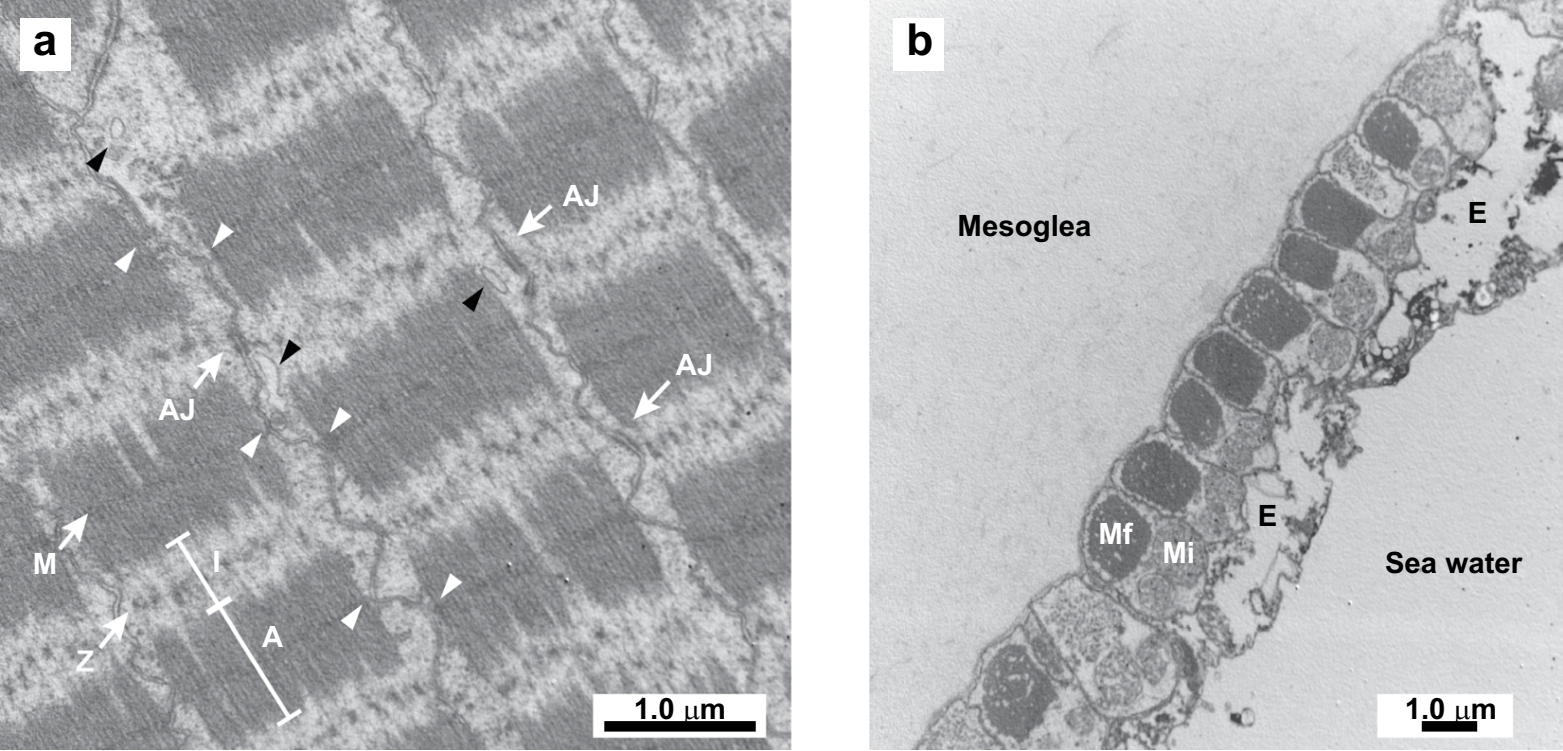
Electron micrograph of adult moon jelly striated muscle. (**a**) Longitudinal section of myofibrils at the plane of the muscular sheet. The A-band (A), I-band (I), M-line (M), Z-disc (Z), and adherence junctions (AJ) are indicated. White arrowheads indicate the point where the M-lines are attached to the inner cell surface. Black arrowheads indicate the vesicles. (**b**) Radial section of subumbrella. The striated myofibrils (Mf), mitochondria (Mi), and epithelial cells (E) are indicated.

The longitudinal section of myofibrils at the plane of the muscular sheet in the subumbrella of *Aurelia* sp. showed features typical of striated muscle (Fig. 1a). However, the sarcomere length, namely, the distance between two adjacent Z-discs, was 1.47 ± 0.02 μm (mean ± standard error; n = 23), which is shorter than those of vertebrate striated muscles (~2.4 μm), but longer than has been previously reported for the same species (0.8 μm^11^ or 1 μm^13^). The length of the A-band (i.e., length of the thick filament) was 0.95 ± 0.01 μm (n = 24). As reported previously^11^, the Z-discs were rather discontinuous and looked fragile, suggesting that the components comprising the Z-disc differ from those of other animals. On the one hand, the M-lines appeared to attract the plasma membrane inward, suggesting that they bind to the inner cell surface and transmit the contractile force to the outside to bring about the shortening of the cell body. On the other hand, there were adherens junctions between the Z-discs of two neighboring myocytes, suggesting that the Z-discs are linked to cell–cell adhesion. Blanquet and Riordan reported similar Z-disc-linked lateral adhesion, describing the structures as desmosomal junctions, in *Cassiopea xamachana* striated muscle^16^. Therefore, the Z-disc is likely to play a role in transmitting the force to other myocytes and to contribute to synchronized contraction of the entire subumbrella. In addition, we observed some small vesicles near the Z-discs. Lin and Spencer proposed that similar vesicles in *Polyorchis penicillatus* contain stores of Ca^2+^ for activating muscular contraction^20^.

The radial section of the umbrella showed a transverse section of the circular striated myofibrils (Fig. 1b). As reported previously^13^, the myoepithelium is composed of three layers: namely, the striated myofibrils, mitochondria/nuclei of myocytes, and epithelial cells layers. In our observations, the cytoplasm of the epithelial cells was sparse, and occasionally there were large vacuoles or voids between the epithelial and myocytes layers. It has been reported that the surface of the subumbrella is partly or completely covered by radially arranged smooth muscle cells in Hydrozoa but not in Scyphozoa (including *Aurelia*) and Cubozoa^14^. We confirmed that neither thick nor thin filaments were present other than the striated myofibrils. Therefore, the native actomyosin fraction that we prepared from the myoepithelium detached from the mesoglea (extracellular gelatinous matrix) should not contain any proteins from smooth muscle tissues, although it may be contaminated with a small amount of non-muscle actomyosin derived from epithelial cells.

### Contractile protein components of native striated actomyosin and expression analysis

**Figure 2:**
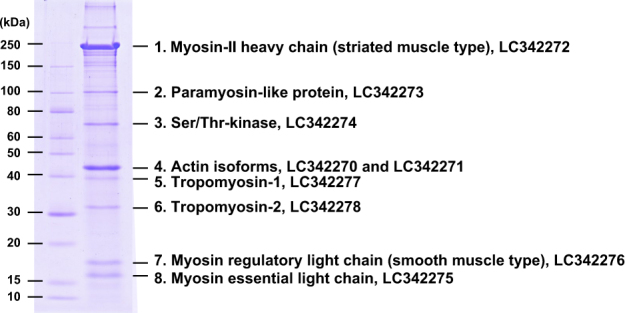
SDS-PAGE of the native actomyosin fraction prepared from the striated muscle of adult *Aurelia* sp. The actomyosin fraction (8 μg) was applied to a 10% acrylamide gel. Eight major bands (1 to 8) of proteins were identified as indicated. The accession numbers of the nucleotide sequences of cDNA encoding each protein are shown.

Our attempts to prepare actomyosin from the homogenate of whole ephyra, umbrella, or frozen subumbrella were unsuccessful. To obtain functional actomyosin, it was essential to use the detached myoepithelium as the starting material. Figure 2 shows the SDS-PAGE separation of the native actomyosin fraction prepared from the striated myoepithelium of adult moon jelly. Eight major protein bands were identified by in-gel protease digestion, followed by mass spectrometry and a Mascot search of the *Aurelia* transcriptome database constructed by Fuchs *et al*.^21^ (data set ‘db454_aaur_rc_final_contigs’ was downloaded from http://www.compagen.org/aurelia/datasets.html). The detailed identification data, including identification scores, are summarized in Supplementary Table S1. On the basis of the nucleotide sequences in the database, we cloned and sequenced the full-length translational regions encoding each protein from the umbrella of adult moon jelly.

**Figure 3:**
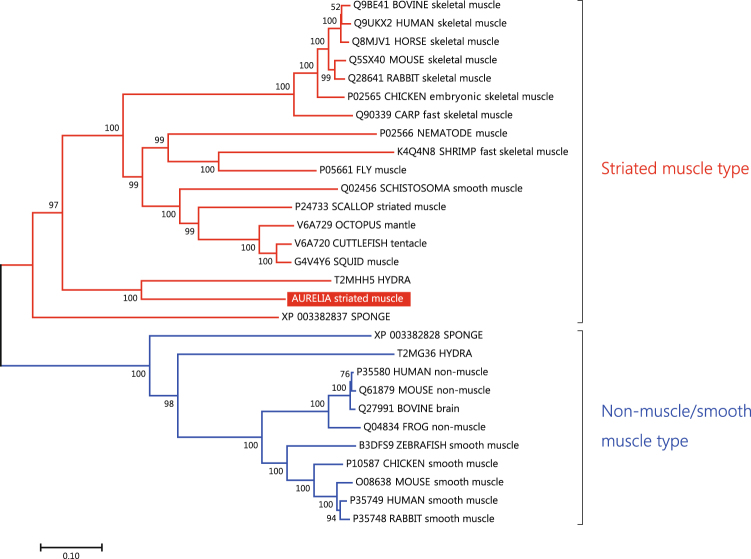
Neighbor-joining analysis of myosin-II heavy chain (MHC-II) proteins. Bootstrap values in percentages are shown on the branch lines. Scale bar represents 0.1 substitutions per site. The accession numbers of amino acid sequences in the UniProtKB/TrEMBL or GenBank (for sponge MHC-II) database are shown. Tissues, organs, or body parts in which the expression of these proteins had been experimentally confirmed are represented by lower case letters.

**Figure 4:**
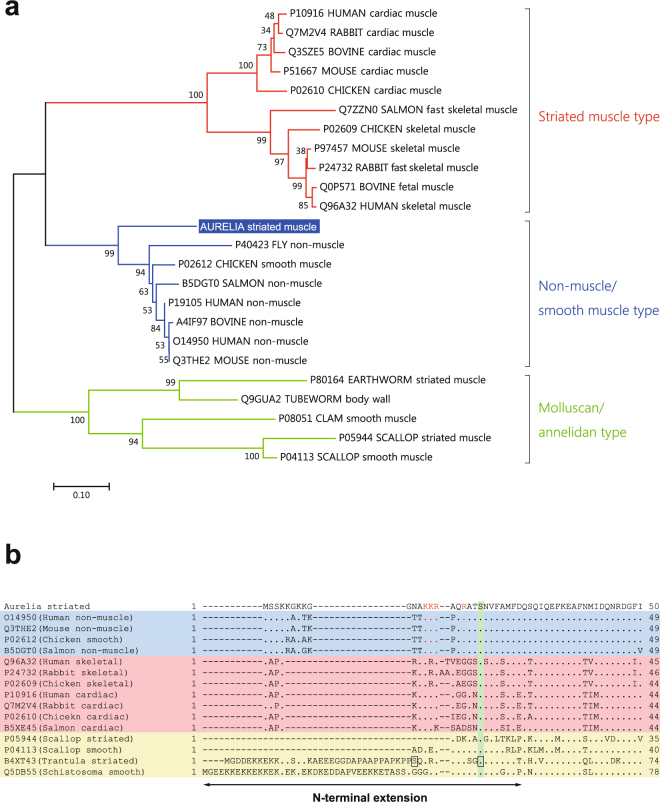
Neighbor-joining analysis of myosin regulatory light chain (MRLC) proteins. (**a**) Neighbor-joining tree generated with amino acid sequences of MRLC proteins. The accession numbers of amino acid sequences in the UniProtKB/TrEMBL are shown. (**b**) Comparison of N-terminal sequences. The N-terminal extension (NTE) is indicated. Residues identical to those of the *Aurelia* MRLC are represented by dots. The serine residues known to be primarily phosphorylated by myosin light chain kinase (MLCK) in vertebrate smooth muscles are shaded in green. The sequences of the non-muscle/smooth, striated, and invertebrate MRLC are shaded in blue, red, and yellow, respectively. Ser35 and Ser45 of tarantula striated muscle MRLC, which are reported to be phosphorylated, are boxed. The basic four residues important for the substrate recognition by smooth muscle MLCK are indicated in red font.

**Figure 5:**
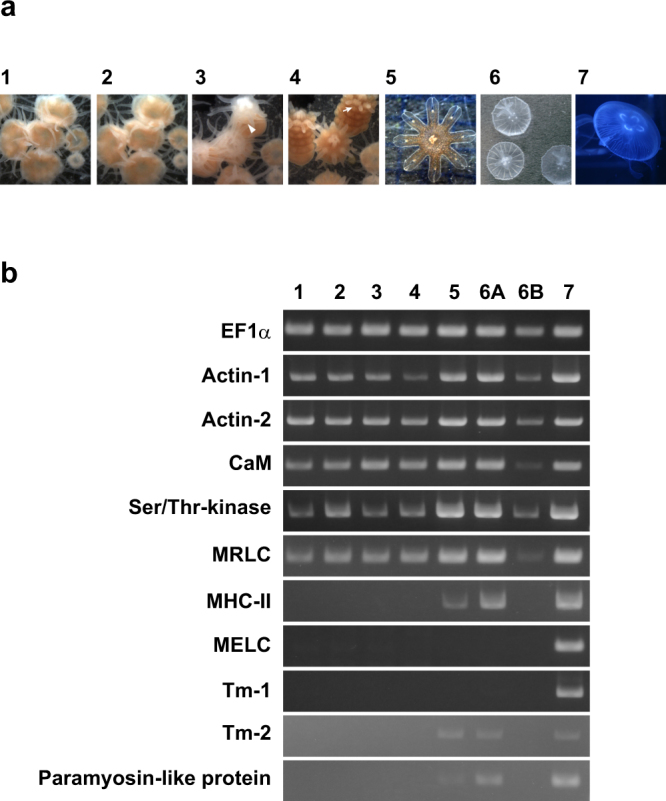
Expression analysis by endpoint RT-PCR. (**a**) Samples used for analysis. 1, Polyps cultivated at 25 °C. 2, Polyps after incubation for 7 days at 15 °C. 3, Early strobila (after 23 days); arrowhead indicates transversal segmentation. 4, Late strobila (after 29 days); the rudiment of the sensory organ (arrow) has developed. 5, Liberated ephyra (after 34 days). 6, Juvenile medusa. 7, Adult jellyfish. (**b**) Qualitative RT-PCR performed on the samples. Whole-animal bodies of 5, 5, and 50 individuals of polyp (1 and 2), strobila (3 and 4), and ephyra (5) were used for analysis. The umbrella (6 A) and oral arms (6B) of a single juvenile medusa were separately analyzed, whereas only the umbrella of adult medusa (7) was analyzed. Prior to using the umbrellas as samples, the marginal part including smooth muscle tissues was removed. Expression of elongation factor 1α (EF1α) gene was measured as a positive control. The images have been cropped from different parts of the gel or different gels. The full-length gels, including DNA size markers, are presented in Supplementary Fig. S4 together with the photographic conditions. Abbreviations: CaM, calmodulin; MRLC, myosin regulatory light chain; MHC-II, myosin-II heavy chain; MELC, myosin essential light chain; Tm, tropomyosin.

Band 1 was identified as the myosin-II heavy chain (MHC-II), whereas bands 7 and 8 were the myosin regulatory light chain (MRLC) and myosin essential light chain (MELC), respectively. The myosin superfamily is composed of many classes, and the myosin-II class includes the conventional myosin that constitutes the muscular thick filament^22,23^. Moreover, MHC-IIs are categorized into two types, namely, a striated muscle-type and non-muscle/smooth muscle-type^18,22–27^, and both types are generally expressed even in primitive animals such as Porifera, which do not have musculature^18,25,27^. Phylogenetic analysis indicated that the moon jelly MHC-II clearly belongs to the cluster of striated muscle-type MHC-IIs (Fig. 3). On the other hand, the MRLC shows higher amino acid sequence similarity to those of vertebrate smooth muscles (75% identity with chicken smooth muscle MRLC) than to those of skeletal muscles (52% with chicken fast skeletal MRLC). Our phylogenetic analysis suggested that there are three major groups of MRLC: (1) vertebrate striated muscle MRLC; (2) non-muscle/smooth muscle MRLC and jellyfish striated muscle MRLC; and (3) molluscan and annelidan MRLC (Fig. 4a). The N-terminal extension (NTE) of MRLC is known to be functionally important and diversified between vertebrates and invertebrates. In the NTE of moon jelly MRLC, Ser21 and Thr20, corresponding to the respective primary and secondary phosphorylation sites in smooth muscle contraction^28^ are conserved (Fig. 4b). In addition, four basic residues, namely, Lys13, Lys14, Arg15 and Arg18, involved in substrate recognition by smooth muscle MLCK^29^, are also conserved. As a result, the NTE of moon jelly MRLC is highly homologous to those of vertebrate non-muscle/smooth muscle MRLC. In some invertebrate muscles, the NTE is extended by up to ~70 residues^26^. For instance, tarantula striated muscle, which is mainly regulated by troponin but also modulated by MRLC phosphorylation, contains a long NTE including two Ser residues: Ser45, which corresponds to Ser21 of the moon jelly MRLC and is phosphorylated by endogenous MLCK; and Ser35, which is in part constitutively phosphorylated, probably by protein kinase C^30,31^ (Fig. 4b). In addition, *Schistosoma* body wall muscle, which is morphologically smooth muscle and probably solely regulated by MRLC phosphorylation, also contains MRLC with a long NTE containing the phosphorylation site for smooth muscle-type MLCK^26^. As described above, the moon jelly striated muscle MRLC has a short NTE and a smooth muscle-type MLCK recognition sequence, and thus is different from these invertebrate MRLCs. Consequently, jellyfish myosin in the striated muscle seems to be a hybrid containing striated muscle-type MHC-II and smooth muscle-type MRLC. It should be noted that *Schistosoma* smooth muscle is composed of the smooth muscle-type thin filament, which lacks regulation by troponin, and the striated muscle-type thick filament, which includes a similar hybrid myosin comprising striated-type MHC-II and smooth-type MRLC with a long NTE^26^. Expression analysis (Fig. 5) indicated that the striated muscle-type MHC-II is expressed only in the ephyra and in the umbrellas of juvenile and adult medusae, suggesting that it is specific for striated muscle. As reported previously, Cnidaria has both striated and non-muscle/smooth muscle-type MHC-IIs^18^. Therefore, the non-muscle/smooth muscle MHC-II isoform of *Aurelia*, which was not identified in this study, is likely to be expressed in the smooth muscle tissues of polyps and the oral arms of medusae. By contrast, expression of the MRLC was detected at all developmental stages including the polyp, suggesting that the MRLC also is likely to be a component of myosin in both smooth muscle and non-muscle tissues. In addition, expression of the MELC was detected in adult but not juvenile medusa, suggesting that some MELC isoforms are differentially expressed in juvenile and adult medusae.

The band 2 protein was found to share less than 30% sequence identity with the C-terminal rod region of MHC-II from other species, but it was not a degradation product of the band 1 protein. The whole protein is predicted to constitute an α-helical coiled-coil (Supplementary Fig. S1), and shares low sequence identity (~23%) with paramyosin from several invertebrates. Therefore, this protein may be a paramyosin-like protein, although previous results of genome or transcriptome mining suggested that Cnidaria lacks paramyosin^18^. On the one hand, paramyosin is known to constitute the core of the thick filament, which sometimes has a large diameter in invertebrate smooth muscles^2^. On the other hand, paramyosin has also been found in non-muscle tissues of invertebrates^32^. Therefore, this protein may not be complexed with the thick filament. The expression of this protein was restricted to the moon jelly stages and tissues containing striated musculature (Fig. 5).

The band 3 protein was identified as a Ser/Thr-kinase. The protein comprises 563 residues and contains two immunoglobulin C2-set domains in the N-terminal region and a kinase domain at the C-terminus, and shows high homology (~60% identity) to *Hydra vulgaris* MLCK (Supplementary Fig. S2). Therefore, the band 3 protein may phosphorylate the smooth muscle-type MRLC described above and is likely to play a key role in regulating the striated muscle contraction. However, this cnidarian kinase lacks an auto-inhibitory region and the binding site for Ca^2+^/CaM. In other MLCKs, the auto-inhibitory region is a pseudosubstrate that mimics the N-terminal phosphorylatable region of the MRLC, and binds to and inhibits the active site of the catalytic domain in the absence of Ca^2+^. In the presence of Ca^2+^, the Ca^2+^/CaM complex binds to the CaM-binding site adjacent to the auto-inhibitory region, and releases it from the active site, resulting in phosphorylation of the MRLC and muscle contraction^29,33^. These regions are conserved in bilaterian MLCKs, but are not likely to be functional in Cnidaria owing to non-conservative amino acid substitutions (Supplementary Fig. S2). Thus, at present, we cannot deduce the mechanism underlying the Ca^2+^-dependent activation of cnidarian kinases. Moreover, this kinase may phosphorylate Ser/Thr residues other than Ser21 of the MRLC, independent of Ca^2+^. It has been reported that such constitutive phosphorylation of Ser35 in the NTE of tarantula MRLC is important for priming the interaction with the Ca^2+^-activated thin filament^31^. Expression of the band 3 protein was observed at all animal stages, similar to actin, CaM, and MRLC (Fig. 5). Therefore, this kinase may be present ubiquitously and involved in many cellular processes including smooth muscle contraction.

The band 4 protein was found to be actin. We identified two actin isoforms, actin-1 and actin-2, the amino acid sequences of which are identical to each other except for three residues near the N-terminus. These isoforms must be encoded by different genes, however, because nucleotide differences were evenly distributed around the translational regions. Both proteins share 92% amino acid identity with human skeletal α-actin, and both were found to be expressed ubiquitously at all developmental stages (Fig. 5).

The band 5 and 6 proteins were identified as tropomyosin isoforms comprising 242 and 246 residues and sharing 42% and 46% identity with tropomyosin-1 and tropomyosin-2, respectively, of the hydrozoan jellyfish *Podocoryna carnea*^34^. The two moon jelly tropomyosin isoforms share only 23% amino acid identity with each other. The molecular masses of tropomyosin-1 and tropomyosin-2 were calculated as 28,049 and 28,741, respectively; however, tropomyosin-1 showed a mass of ~39 kDa on SDS-PAGE (Fig. 2). We confirmed that recombinant tropomyosin-1 prepared by an *Escherichia coli* expression system constructed with the full-length translational region of the cloned cDNA showed similar anomalous mobility (Supplementary Fig. S3). Gröger *et al*.^34^ reported that tropomyosin-1 of *P. carnea* is a non-muscle isoform and is expressed in all tissues, whereas tropomyosin-2 is specific to the striated muscle of the subumbrella. On SDS-PAGE (Fig. 2), however, the tropomyosin-1 and tropomyosin-2 bands appeared to show similar staining intensity, suggesting that they are both present in almost equal amounts in moon jelly striated muscle. Expression analysis indicated that both tropomyosin isoforms are expressed in the umbrella of medusae but not in oral arms or polyps.

Collectively, it was confirmed that troponin is not present in moon jelly striated muscle. However, moon jelly MRLC was found to be highly homologous to the MRLC of smooth muscle and there was a significant amount of a Ser/Thr-kinase resembling MLCK, suggesting that muscle contraction in moon jelly striated muscle may be regulated by Ca^2+^-dependent phosphorylation of the MRLC, as in the case of smooth muscle contraction.

### Biochemical properties of moon jelly striated actomyosin

**Figure 6:**
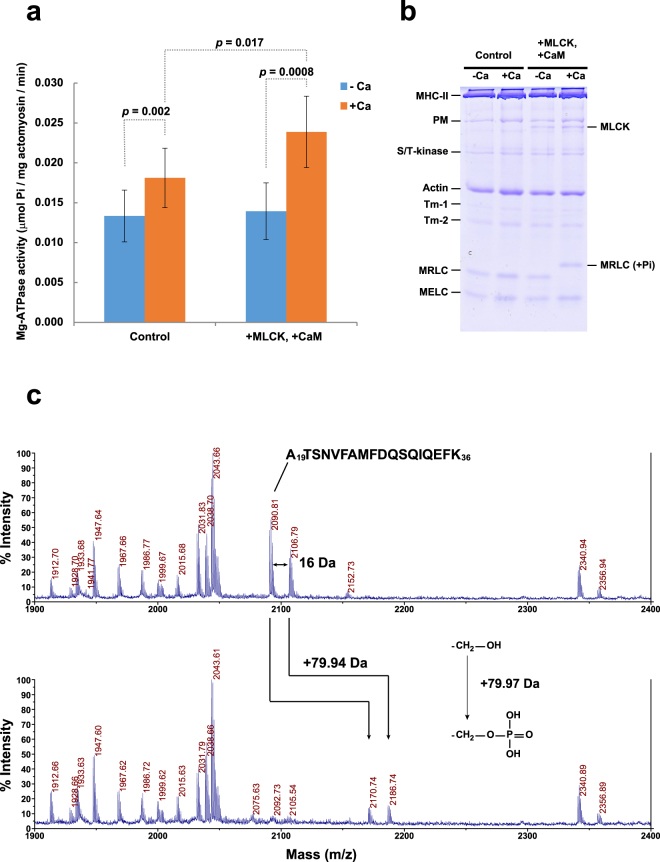
Ca^2+^-dependent activation and phosphorylation of actomyosin. (**a**) Mg-ATPase activity of actomyosin in the absence and presence of Ca^2+^. Ca^2+^-dependent activation was enhanced by adding myosin light chain kinase (MLCK) and calmodulin (CaM). The values were presented as means and standard errors (*n* = 5). The values were also statistically compared by two-tailed *t*-test (*n* = 5), and the *p*-values are indicated. (**b**) Phos-Tag SDS-PAGE of actomyosin incubated for 10 min under the same conditions as the ATPase assay. Abbreviations: MRLC, myosin regulatory light chain; MHC-II, myosin-II heavy chain; PM, paramyosin-like protein; MELC, myosin essential light chain; Tm, tropomyosin. (**c**) Comparison of the mass spectra of tryptic digests of unphosphorylated (upper) and phosphorylated (lower) myosin regulatory light chain (MRLC). The mass increment of 79.94 Da corresponds to phosphorylation of a single hydroxyl group.

To assess the regulatory mechanism of muscle contraction further, we performed an ATPase assay (Fig. 6a). The Mg-ATPase activity of the native moon jelly actomyosin fraction was weakly activated in a Ca^2+^-dependent manner. By adding chicken gizzard MLCK and recombinant moon jelly CaM, the ATPase in the presence of Ca^2+^ was further activated, resulting in an enhancement of the Ca^2+^-dependent actin–myosin interaction. Under similar conditions, Ca^2+^-dependent phosphorylation of the MRLC was observed by Phos-Tag SDS-PAGE (Fig. 6b). In-gel tryptic digestion of the bands of phosphorylated and unphosphorylated MRLC revealed that the peptide A_19_TSNVFAMFDQSQIQEFK_36_ (m/z = 2090.8), and the same peptide with oxidized Met (m/z = 2106.8), showed a 79.9-Da increase in mass in the presence of Ca^2+^ (Fig. 6c), suggesting that Ser21, corresponding to the phosphorylatable Ser20 residue of vertebrate smooth muscle MRLC (Fig. 4b), was phosphorylated. These results indicated that a smooth muscle-like regulatory mechanism may function in moon jelly striated muscle. Notably, however, the Ca^2+^-dependent activation in the presence of MLCK and CaM was weak, even though the MRLC seemed to be completely phosphorylated in the presence of Ca^2+^ (Fig. 6b). Moreover, the slight Ca^2+^-sensitivity of the ATPase activity in the control experiment did not correlate with phosphorylation, suggesting that another regulatory mechanism independent of MRLC phosphorylation may also be present. One possibility is that Ca^2+^ is directly bound to the light chains of myosin and activates the actin–myosin interaction, as in the case of molluscan muscle; however, the presence of this mechanism would bring about more prominent Ca^2+^-dependent activation of the ATPase, because a large amount of both actin and myosin are present in the actomyosin fraction. Another possibility is that the Ser/Thr-kinase binds actin or myosin independent of kinase activity and inhibits or activates the actin–myosin interaction. It has been reported that similar interactions in vertebrate smooth muscle MLCK are affected by Ca^2+^/CaM^35^. Therefore, the latter mechanism might be actuated by the trace amount of CaM present due to contamination from the cytosol in the control experiment.

In the ephyra stage of moon jelly, and even in the adults of many other jellyfish species, the pulsation rate reach more than one pulse per second^36–39^. Such movements require rapid contraction and relaxation of the subumbrella muscle. Therefore, a smooth muscle-like regulatory mechanism, which depends on phosphorylation and dephosphorylation mediated by the sequential association and dissociation of Ca^2+^, CaM, MLCK and myosin phosphatase, which is distributed in the cytosol, may not be appropriate. Many other invertebrate muscles that have fast contraction and relaxation properties, such as adductor muscles of bivalves^2,7^, insects flight muscles^2,40^, and the cercarial striated muscle of *Schistosoma*^26,41^, are considered to be regulated mainly or partly by the troponin system including direct binding of Ca^2+^ to the thin filament. However, troponin subunits are absent from the cnidarian genome, and we confirmed this at the protein level. Thus, it is likely that another unknown regulatory mechanism for cnidarian muscle contraction that does not depend on phosphorylation/dephosphorylation may be acting in concert with a smooth muscle-like mechanism.

## Materials and Methods

### Jellyfish

Adult moon jellies, *Aurelia* sp. (15–25 cm in diameter), were captured at the port of Esashi, Hokkaido prefecture, Japan, or in some cases, were kindly provided from Kamo aquarium (Tsuruoka, Japan). To obtain actomyosin, the animals were first anesthetized by submerging them in menthol-saturated artificial sea water (ASW), and then the oral arms, gastric filament, gonad, tentacles, and marginal part (containing smooth muscle^13^) of the umbrella were carefully removed. The remaining umbrella was cut into 3–4 parts and immersed in menthol-saturated ASW containing 10% ethanol for 15–60 min at room temperature. Next, the myoepithelium was detached from the undersurface of the umbrella by scratching with forceps, and was collected by pipetting and centrifugation. The myoepithelium was stored at −20 °C in 50% glycerol, 20 mM KCl, and 5 mM KH_2_PO_4_ (pH 7.0) containing Complete Protease Inhibitor Cocktail (Roche).

Polyps of moon jelly were purchased from an aquatic animal supplier, ‘My-AQUA’ (Nagoya, Japan), and cultivated at 25 °C by feeding with *Artemia salina*. To induce strobilation, the polyps were fasted for 4–6 weeks at 15 °C. The liberated ephyrae were fed at 15 °C and grown to adult medusae (~8 cm in diameter). Animals at each developmental stage—namely, polyp, early strobila (one or two segmentations on body column were observable), late strobila (the color changed to brown and the rudiment of marginal lappet with sensory organ was observable), ephyra, juvenile medusa, and adult medusa—were used for the expression analysis (Fig. 5a) and cDNA cloning.

The genus *Aurelia* is known to include many endemic and cryptic species. PCR amplification and sequencing of the *ITS-I/5.8 S rDNA* regions of genomic DNA by the method of Fuchs *et al*.^21^ indicated that the specimens used in this study were the most globally distributed species, namely, *Aurelia* sp. 1 (*Aurelia coerulea*^42^).

### Electron microscopic observations

The umbrella of an anesthetized adult medusa (8 cm in diameter) was cut into square pieces of 7 × 7 mm and prefixed by intermittent microwave irradiation for 1 min in 4% paraformaldehyde, 2.5% glutaraldehyde, and 0.1 M sodium cacodylate (pH 7.5). The subumbrella part was excised, cut into pieces of 1–2 mm, and then post-fixed with 1% osmium tetraoxide in 0.1 M sodium cacodylate (pH 7.5) for 1 h at room temperature. Next, the pieces were dehydrated by an ethanol series and embedded in Quetol-812 epoxy resin (Nisshin EM). Sections of 70-nm thickness prepared by an ultramicrotome (UMC7, Leica) were adhered to copper grids, and stained first with EM Stainer (Nisshin EM) diluted 4-fold with distilled water, and then with Reynolds’ lead citrate solution^43^. Observation was performed with a transmission electron microscope (JEM-1011, JEOL) at an acceleration voltage of 80 kV.

### Preparation of Aurelia adult striated actomyosin and ATPase assay

The myoepithelium was homogenized in 40 mM KCl, 10 mM KH_2_PO_4_ (pH 7.0), and 0.01% NaN_3_ containing Complete Protease Inhibitor Cocktail for 1 min. The suspension was centrifuged, and the supernatant was discarded. These homogenization and centrifugation steps were repeated once more. The precipitate was dispersed in 0.6 M KCl, 10 mM Tris-HCl (pH 7.6), 0.01% NaN_3_ and 2 mM ATP, and left for 2 h on ice to extract actomyosin. The extract was collected by centrifugation, and diluted with 9 volumes of ice-cold water. The precipitated actomyosin was collected by centrifugation, re-dissolved, and then dialyzed against 0.6 M KCl, 10 mM Tris-HCl (pH 7.6) and 5 mM 2-mercaptoethanol. The SDS-PAGE analysis revealed that the actomyosin fraction was enriched in eight major contractile or regulatory proteins, but contained a small amount of multiple unidentified impurities. The Mg-ATPase activity of the actomyosin fraction was determined as described previously^44^ under the conditions of 0.05 mg/ml of actomyosin, 50 mM KCl, 20 mM Tris maleate (pH 6.8), 2 mM MgCl_2_, 0.2 mM EGTA, 0.3 mM CaCl_2_ (in the presence of Ca^2+^), and 1 mM ATP at 15 °C in the presence and absence of 0.006 mg/ml of chicken gizzard MLCK and 0.007 mg/ml of *Aurelia* recombinant CaM (see below).

### Preparation of Aurelia recombinant proteins

The cDNA encoding *Aurelia* CaM was cloned from the polyp by RT-PCR with AaCaMFw and AaCaMRv primers (Supplementary Table S2), which were based on the nucleotide sequence, ‘03_aurelia_rc_finalASM_10773’, in the *Aurelia aurita* transcriptome database^21^. The cDNA was ligated into the *Nco*I/*Bam*HI sites of pET-16b (Novagen) and transformed into *Escherichia coli* BL21 Rosetta2 (DE3) (Novagen). Cultivation of transformants, induction of protein expression, and purification of recombinant CaM were performed as described for lobster troponin C mutants^44^.

Chicken gizzard MLCK was prepared by the method established by Adelstein and Klee^45^ with the modification of Kondo *et al*.^46^.

### SDS-PAGE and identification of protein by mass spectrometry

The proteins included in the native actomyosin fraction were separated on SDS-PAGE by the method of Laemlii^47^ with the buffer system in the separation gels substituted by 100 mM Tris-300 mM Gly (pH 8.9) based on the modifications of Porzio and Pearson^48^. The samples were run with molecular mass markers (SP-2110, APRO life Science). For the phosphorylation analysis, the separation gels contained 45 μM Phos-Tag acrylamide (Wako pure chemicals) and 90 μM MnCl_2_. The gels were stained with CBB R-250. The major bands were excised, de-stained twice by agitation for 10 min in 50% acetonitrile and 25 mM NH_4_HCO_3_, and then dehydrated in 100% acetonitrile followed by vacuum drying. The dried gels were impregnated with 0.01 mg/ml of trypsin (Promega) or lysylendopeptidase (Wako pure chemicals) dissolved in 25 mM NH_4_HCO_3_, and incubated overnight at 37 °C. The resulting peptides were extracted with 50% acetonitrile and 5% trifluoroacetic acid (TFA), and vacuum dried by using a centrifugal vaporizer. All peptides were desalted with a ZipTip C_18_ column (Merck). In addition, all tryptic peptides and the lysylendopeptidase peptides from bands 2, 5, 6, and 7 in Fig. 2 were bound to the ZipTip C_18_ column and subjected to solid-phase derivatization, namely, the ε-amino groups of lysine residues were guanidinated with *O*-methylisourea, followed by the N-terminal derivatization with 4-sulfophenyl isothiocyanate (SPITC)^49,50^. Peptides eluted from the ZipTip column by 60% acetonitrile and 0.1% TFA were mixed with the same volume of 5 mg/ml of α-cyano-4-hydroxycinamic acid, 50% acetonitrile, and 0.1% TFA, dried on a stainless target plate, and then analyzed with a MALDI-TOF mass spectrometer (4700 proteomics analyzer, ABSciex). To identify proteins, the spectra obtained in positive ion reflector mode and the MS/MS spectra of the major peptide ions observed were subjected, respectively to peptide mass fingerprinting (PMF) and MS/MS ions search (MIS), using the transcrisptome database^21^ registered on a local Mascot server (Version 2.2.07, Matrix Science). The following parameters were used: allowance of one (trypsin) or no (lysylendopeptidase) missed cleavage; mass tolerance, ±0.4 Da; peptide charge, 1+; and fixed Guanidinyl (K) modification (for the spectra of guanidinated samples). *De novo* sequencing of the peptides from the MS/MS spectra of the SPITC derivatives was also performed by using the method of Chen *et al*.^50^. The sequences identified in the transcriptome database were subjected to a BLAST search on the NCBI-nr database for annotation.

### cDNA cloning and expression analysis

Total RNA was prepared by using RNA Iso Blood (Takara bio) in accordance with the manufacturer’s instructions with some modifications. Samples were added to 1 ml of the reagent and homogenized with a pestle; 200 μl of chloroform was then added, followed by intense mixing. After centrifugation, the aqueous layer (~0.3 ml) was separated and mixed with 2-propanol (0.15 ml) and High-salt Solution for Precipitation (Plant) (0.15 ml) (Takara bio) to precipitate RNA. The precipitated total RNA (200 ng) was used as a template for cDNA synthesis by using a PrimeScript II 1st strand cDNA Synthesis Kit (Takara bio) with the primer *Not*I-dT_18_ (Supplementary Table S2). The resulting cDNAs were used as the template for RT-PCR. The PCR primers used in this study are summarized in Supplementary Table S2. We also performed 3′- and 5′-RACE to obtain the full-length cDNA. The PCR products were subcloned into pCR2.1-TOPO (Invitrogen) and sequenced with a BigDye Terminator v3.1 Cycle Sequencing Kit and 3130XL genetic analyzer (Applied Biosystems). The nucleotide sequences of the translational regions of MHC-II, paramyosin-like protein, Ser/Thr-kinase, actin-1, actin-2, tropomyosin-1, tropomyosin-2, MRLC, and MELC were registered in the DDBJ database with accession numbers LC342272, LC342273, LC342274, LC342270, LC342271, LC342277, LC342278, LC342276, and LC342275, respectively. In the expression analysis, expression of the elongation factor-1α (EF1α) gene was analyzed as a positive control by using the primer set aEF1_FII and aEF1_RII described by Fuchs *et al*.^21^.

### Phylogenetic analysis

Amino acid sequences of MHC-II and MRLC were obtained from the UniProt database. We collected only complete sequences of the proteins. The existence of all proteins has been experimentally confirmed at the protein or transcript level, except for the sponge MHC-II isoforms, the amino acid sequences of which have been predicted from the genomic sequence by automated analysis. Multiple alignments were prepared by the MUSCLE^51^ algorithm in Genetyx-Win Ver. 13 (Genetyx) with default settings, and the results were used to construct neighbor-joining phylogenetic trees by using MEGA7^52^ with bootstrap replication set at 1000.

### Equipment and settings

The PCR products were run on 1% agarose gels in 40 mM Tris acetate (pH 8.3) and 1 mM EDTA containing 0.5 μg/ml ethidium bromide. The gels were photographed by using a transilluminator (MBP-UV312JP, Mecan imaging) with a conventional digital camera (EOS kiss X7, Canon) equipped with an EF-S18–55 mm IS II lens (Canon) and SC58 filter (Fujifilm).

The Mg-ATPase activity data for five different preparations of actomyosin were collected. Values in the absence and presence of Ca^2+^ were statistically compared by two-tailed Student’s *t*-test in Microsoft Excel 2013.

### Data availability

Data supporting the findings of this study are available from the corresponding author on reasonable request.

## Electronic supplementary material

Supplementary information
