# Supplementary material for: Smooth muscle-like Ca2+-regulation of actin–myosin interaction in adult jellyfish striated muscle
Source: Sci Rep. 2018 May 17;8:7776. doi: 10.1038/s41598-018-24817-x (PMC5958069; doi:10.1038/s41598-018-24817-x)
Supplement: Supplementary file 1 — Supplementary information [file 41598_2018_24817_MOESM1_ESM.pdf]

Supplementary information

Smooth muscle-like Ca^{2+}-regulation of actin–myosin interaction in adult jellyfish striated muscle

Hiroyuki Tanaka*, Shiori Ishimaru, Yasuhiro Nagatsuka, and Keisuke Ohashi

Laboratory of Marine Biotechnology and Microbiology, Graduate School of Fisheries Sciences, Hokkaido University, Hakodate, Japan

*Corresponding author: Hiroyuki Tanaka

Laboratory of Marine Biotechnology and Microbiology,

Graduate School of Fisheries Science,

Hokkaido University,

3-1-1, Minato-cho, Hakodate, Hokkaido 041-8611, Japan

E-mail: tanaka-h@fish.hokudai.ac.jp

Tel: +81 138 40 8809

Supplementary Table S1 Summary of the identification of protein components constituting actomyosin prepared from striated muscle of *Aurelia* sp.

Band	Protein[a]	Accession ID. in *Aurelia* transcriptome database[21]	In gel tryptic digestion				In gel lysylendopeptidase digestion				*De novo* sequencing[d]
			unmodified		guanidinated		unmodified		guanidinated		
			PMF[b]	MIS[c]	PMF	MIS	PMF	MIS	PMF	MIS	
1	Myosin-II heavy chain	03_aurelia_rc_finalASM_8407	118		185	(6) 163>34					
2	Paramyosin-like protein	03_aurelia_rc_ephyra_run091013_8549		(1) 67>35		(2) 54>35			80		
		03_aurelia_rc_ephyra_finalASM_3359					83	(2) 40>31		(3) 79>31	
		03_aurelia_rc_ephyra_finalASM_636						(1) 35>31		(1) 54>31	
3	Ser/Thr-kinase	03_aurelia_rc_finalASM_6890	89	(4) 149>36	108						
4	Actin	03_aurelia_rc_finalASM_28	134		113	(2) 88>36					
		03_aurelia_rc_finalASM_3921	127		103	(2) 88>36					
		01_aurelia_rc_polyp_run090924_535	103		102						
5	Tropomyosin-1	03_aurelia_rc_finalASM_6847		(2) 189>34			75	(3) 115>28	91		S(L/I)ENRVK
		03_aurelia_rc_finalASM_6846		(2) 189>34				(3) 115>28	77		
		03_aurelia_rc_finalASM_7777		(2) 160>34				(2) 108>28			
6	Tropomyosin-2	03_aurelia_rc_finalASM_8686	74	(3) 177>36	98		104	(4) 116>29	125	(4) 104>31	(L/I)(L/I)E(L/I)E(L/I)ENYK, H(L/I)(L/I)EE,
7	Myosin regulatory light chain	03_aurelia_rc_finalASM_12112		(1) 73>35			81	(3) 116>29	71	(5) 241>30	QNRDGF(L/I)DK, EDY(L/I)D, AD(L/I)QDMYAS(L/I)GK, NYVEFTR
		03_aurelia_rc_finalASM_12111		(1) 73 > 35			79	(3) 116>29	69	(5) 241>30	
		01_aurelia_rc_ephyra_run090924_5376					77		68		
		03_aurelia_rc_finalASM_8799		(1) 73>35				(3) 116>29			
		03_aurelia_rc_finalASM_8800		(1) 73>35				(3) 116>29			
8	Myosin essential light chain	03_aurelia_rc_finalASM_812		(2) 112>36		(2) 85>35					

a Final identification based on the results of a BLASTP search of the NCBI-nr database using the amino acid sequences identified in the *Aurelia* transcriptome database as queries.
b Peptide mass fingerprinting. Values are identification scores greater than 68, suggesting significant identification (P <0.05).
c MS/MS ions search. Values in parentheses are the numbers of precursor ions used for identification. Identifications that yielded scores greater than the thresholds indicated were considered to be significant (P <0.05).
d Amino acid sequence determined directly from the MS/MS spectrum of the peptide by the method of Chen *et al.*[50]. Leu and Ile could not be distinguished because their molecular masses are identical.

Supplementary Table S2 Sequences of primers used in the study

Target	Name	Sequence	Purpose[a]
MHC-II	AaMHCFw	AGCAAGAGATCGGCAAACTACG	I, E
	AaMHCRv	CAGCAAAGTCTGATTCGGCCT	I
	AaMHCFw2	ATCAAGAACCAACGTCGCTCGG	3R
	AaMHC5UTRFw	ATCGATGCACAGGGCAGATC	F
	AaMHC3UTRRv	AGTTCGATCTAACTACCAAGGTCC	F
	AaMHCRv2	AGCTCATCTGCGAGATTC	E
Paramyosin-like protein	AaParaMFw	GACGAAGCAAGAATACTACAGG	I, E
	AaParaMRv	GCTCAGATGCTCTCAGTTTATC	I, E
	AaParaMFw2	AGCTTGGCTTCTGCAGAGGA	3R
	AaParaMRT	TGACTTGTTCGCTCATCACTTTGTG	5R, RT
	AaParaMFw3	ACGAGATATGGCAGCAGATC	5R
	AaParaMRv2	TCTTCCAACTGCTCTGCAAG	5R
	AaParaM 5UTRFw	AGTGTGCTTTGCATTAGTCCTG	F
	AaParaM 3UTRRv	ATAAACACACTTCATCATTCAGACAG	F
Ser/Thr kinase	AaSTKFw	ATGATGGTAACCCAAGGCCAG	I, E
	AaSTKFRv	GCCCAAATATACTCTTCCTCTTC	I, E
	AaSTK 5UTRFw	AGGGTCAGGCTTCGAGTCCTTC	F
	AaSTK 3UTRRv	AAGTGAAGGATCCTGTCGTTGAAG	F
Actin	AaActinFw	AAGGACTCGTACGTCGGAG	I
	AaActinRv	GAACATAGTCGTACCTCCAGA	I
	AaActin1 5UTRFw	AAGAAGAAAGAAGTCAAACCTTCG	F, E
	AaActin1 3UTRRv	TGAATTAGCGGACTAGGATCTG	F, E
	AaActin2 5UTRFw	ACTTCGAAACGGCAAGACC	F, E
	AaActin2 3UTRRv	TGAAACGGTTCGAACACATAG	F, E
Tropomyosin-1	AaTm1Fw	ACGCCAAGGCCCAAGCTG	I, 3R
	AaTm1Rv	GCTCCTTAGGTTGTTGCCAACT	I
	AaTm1 5UTRFw	ATTGTGCTCAGTTCGGCAAGTAGC	F, E
	AaTm1 3UTRRv	ATCGAGTTCCAACCAGGAAACAATC	F, E
Tropomyosin-2	AaTm2Fw	TCGACGAAGCAGATAACAGG	I, 3R
	AaTm2Rv	GTGTCGGTGTCCAACATCAC	I
	AaTm2Fw2	AATCTCTCCGCGTCCTCGAAG	3R
	AaTm2 5UTRFw	AAGCTTCGAGTAGCTGACAGC	F, E
	AaTm2 3UTRRv	AGTCCATTTAAACTACAAACAAGGC	F, E
MRLC	AaMRLCFw	TGATCAGTCGCAGATTCAGG	I, E
	AaMRLCRv	TGAACTCTACATAGTTGAAATTGCC	I, E
	AaMRLC 5UTRFw	AGGTTGTGTTGGCCCACTGTATTG	F
	AaMRLC 3UTRRv	TAACAAGATCCTACAGAAAATACCGCC	F
MELC	AaMELCFw	TGGGATTGAATCCTCTCACAGC	I
	AaMELCRv	CCATTGTCCCGAGACCCT	I, E
	AaMELCFw2	ATGGTCGAGGGTCTCGGGACAATG	3R
	AaMELC 5UTRFw	AGCTAGCAGACGGTTTGCTG	F, E
	AaMELC 3UTRRv	ACCTGACCCCTATGCTCAC	F
CaM	AaCaMFw[b]	CAATTTACCATGGCTGATCAATTGACTGAAG	I, E
	AaCaMRv[b]	ATGTCAAAATGGATCCTTTTACTTTGATGTCATC	I, E
	Not I-dT_{18}	AACTGGAAGAATTCGCGGCCGCAGGAATTTTTTTTTTTTTTTTTT	RT
	Not I-anchor	TGGAAGAATTCGCGGCCGCAGG	3R

a Abbreviations: I, initial PCR; E, expression analysis; F, full-length amplification of translational region; 3R, 3'RACE; 5R, 5'RACE;
b A *Nco* I or *Bam* HI site (underlined) was included to construct the expression plasmid.

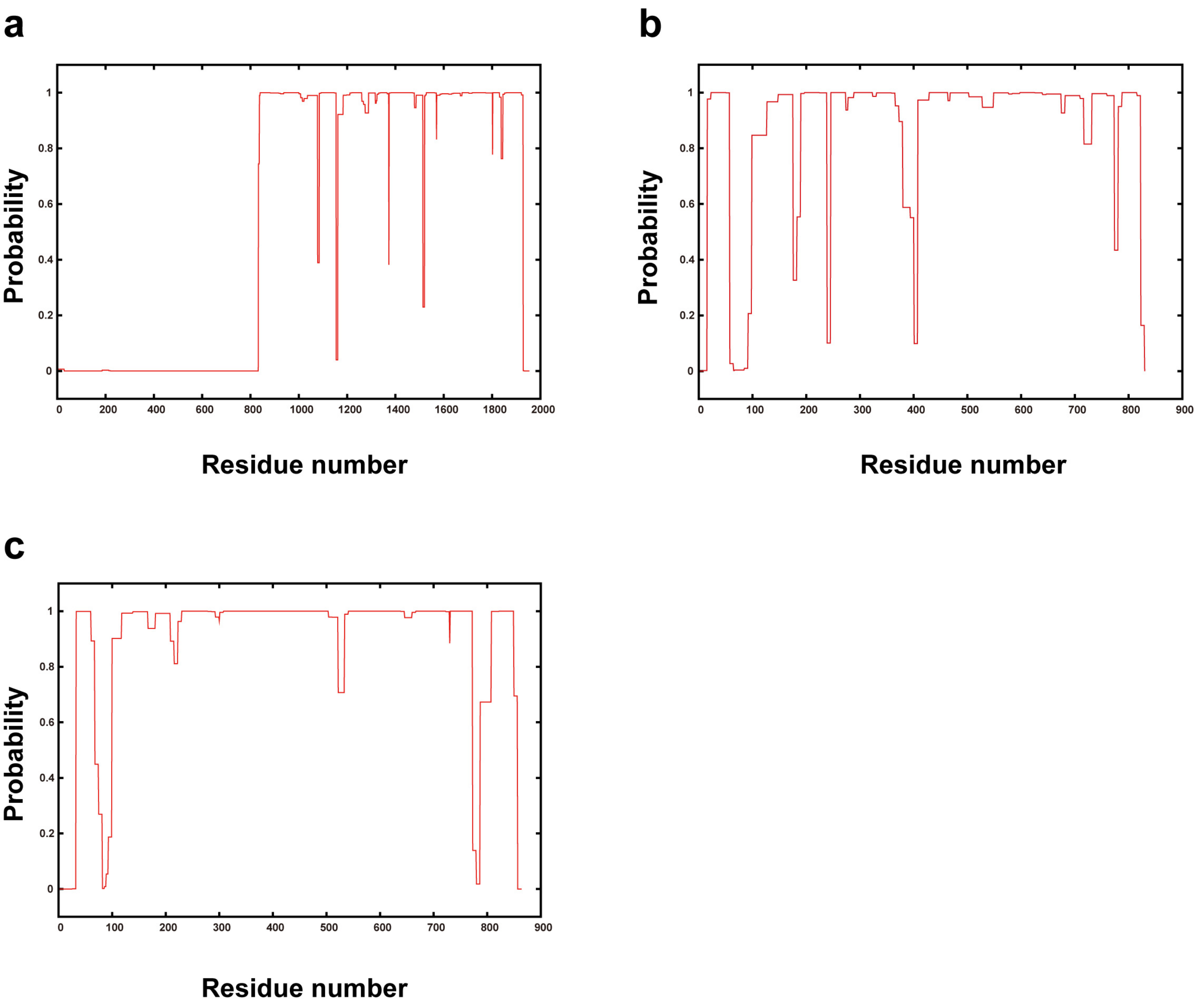

Fig. S1 Prediction of coiled-coil regions by the COILS program[53]. The probability of coiled-coil formation was plotted against residue number. The parameter settings were as follows: scanning window, 28; matrix, MTDIK; no weighting option for highly charged protein. (a) Moon jelly MHC. (b) Moon jelly paramyosin-like protein. (c) Mussel paramyosin (BAA36517).

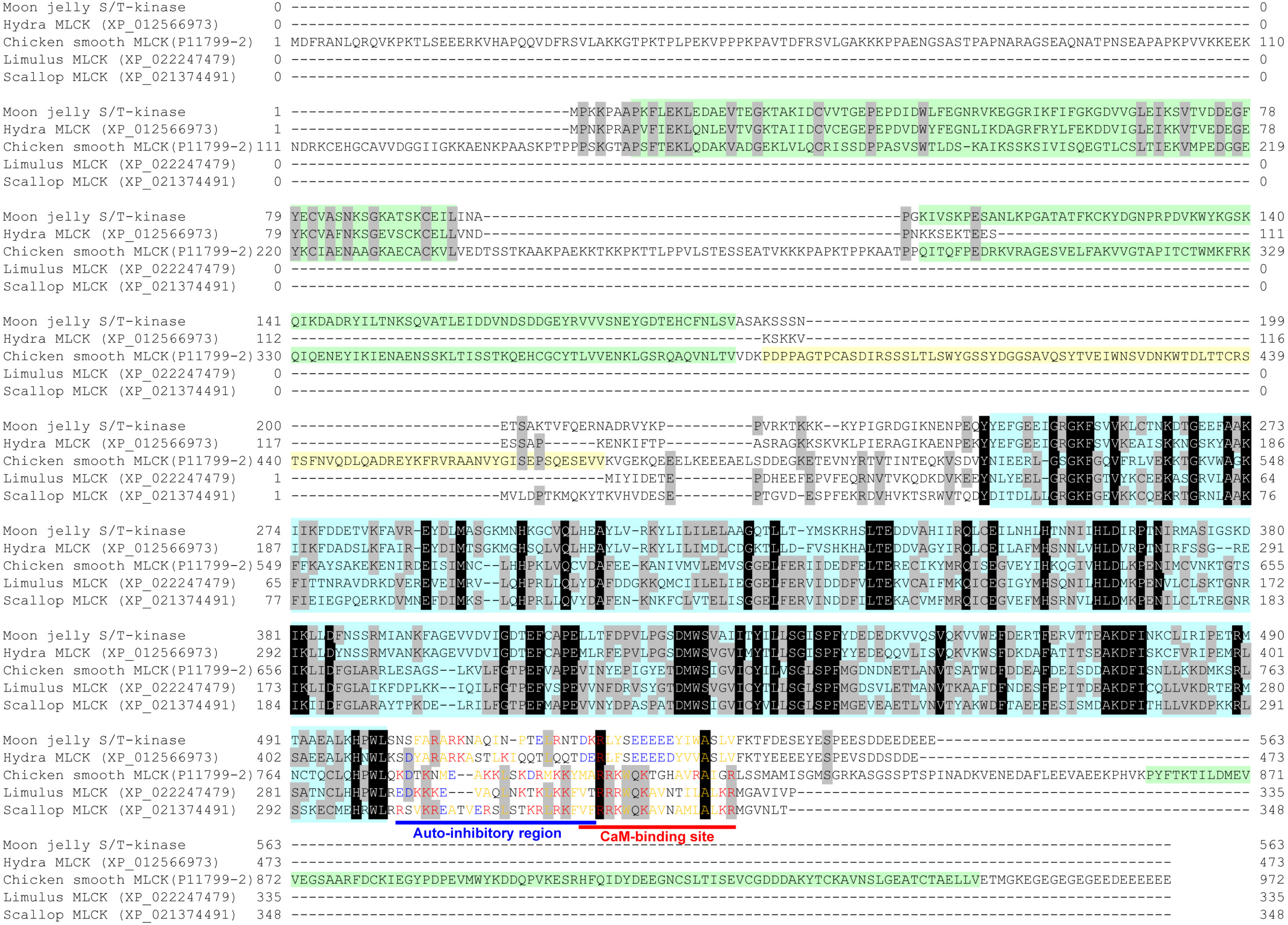

Fig. S2 Multiple alignment of moon jelly Ser/Thr-kinase and myosin light chain kinases (MLCKs). Residues that are identical in all or more than three of the represented proteins are shaded in black or gray, respectively. The immunoglobulin-like motif, fibronectin type-III motif, and the catalytic domain of Ser/Thr-kinase, predicted by a conserved domain search[54] of NCBI' s conserved domain database, are highlighted in green, yellow, and cyan, respectively. Acidic, basic, and hydrophobic amino acids in the auto-inhibitory region and Ca^{2+}/calmodulin binding site are represented by blue, red, and orange characters, respectively.

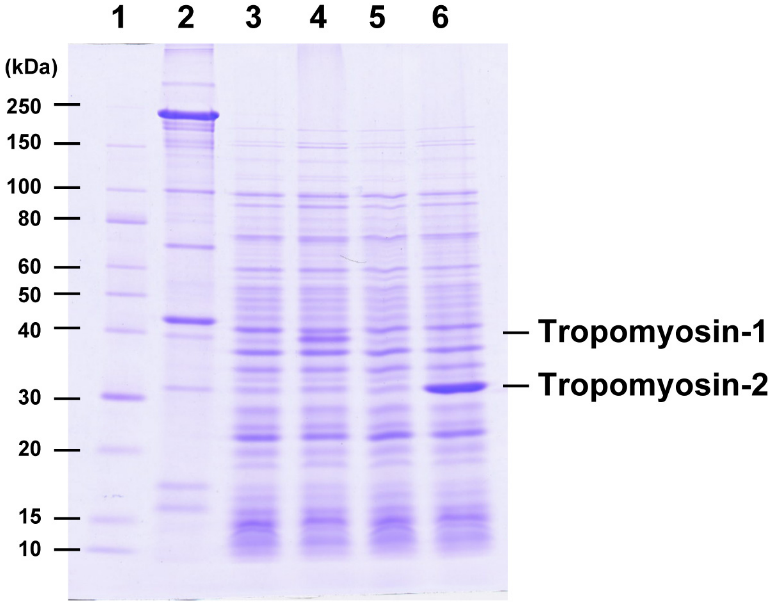

Fig. S3SDS-PAGE of native and recombinant tropomyosin. Lane 1, molecular mass markers; lane 2, native actomyosin (5.4 μg); lane 3 and 4, *E. coli* cell before and after 4h induction of the expression of tropomyosin-1; lane 5 and 6, *E. coli* before and after 4h induction of the expression of toropmyosin-2.

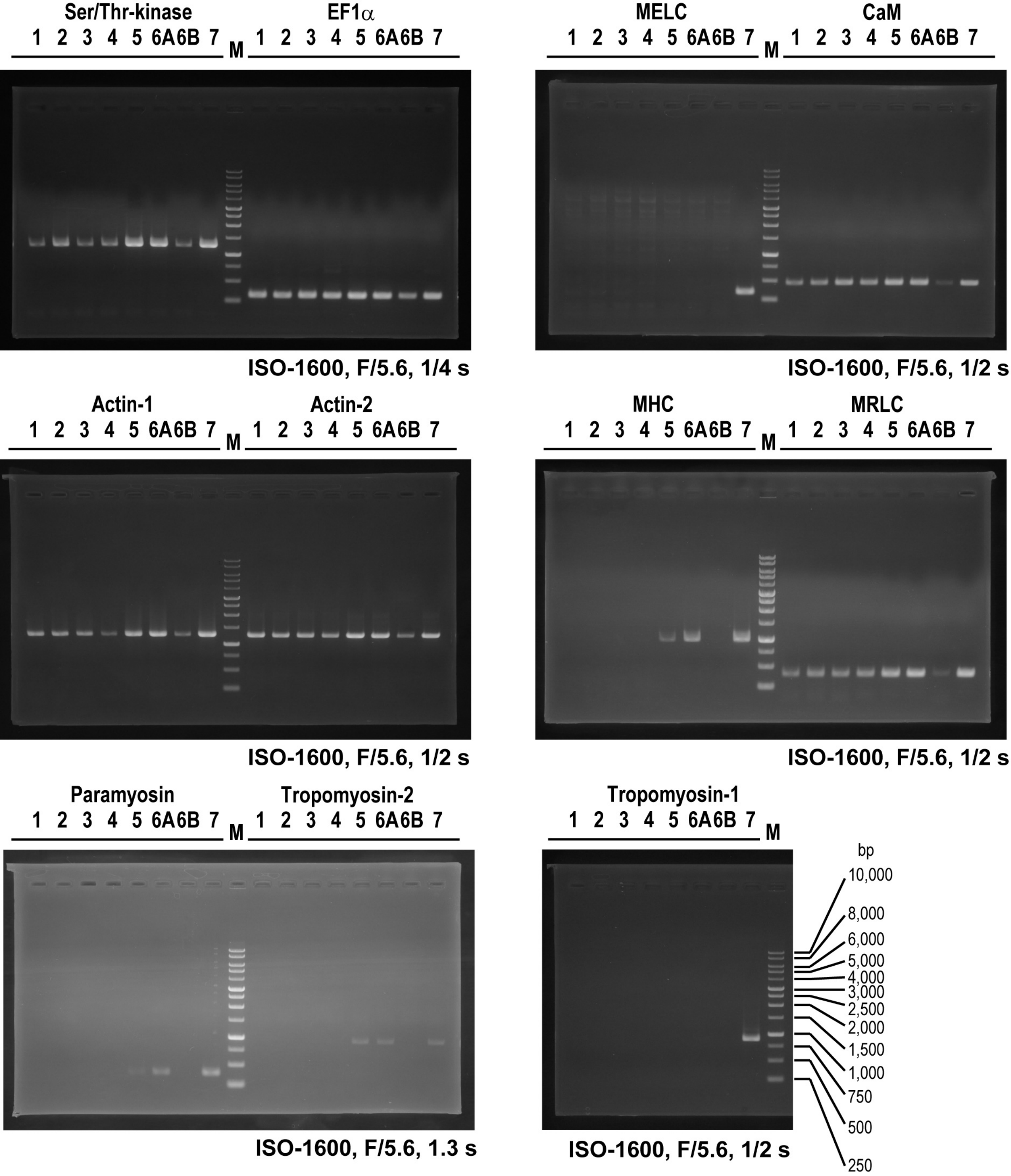

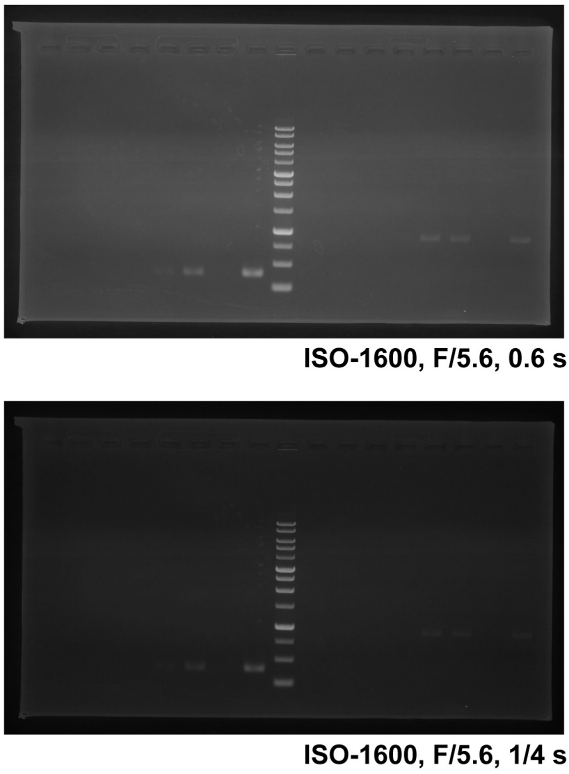

Fig. S4 Agarose gel images used for preparation of Fig. 5b. The photographic conditions (ISO sensitivity, aperture value, and exposure time) are indicated at the bottom left of each image. M indicates DNA size markers (1-kb DNA Ladder RTU, Nippon Genetics). Because the bands for tropomyosin-2 were rather weak, the exposure time was lengthened to 1.3 s, and images obtained with the different two settings are presented.

References

53. Lupas, A., Van Dyke, M. & Stock, J. Predicting coiled coils from protein sequences. *Science* **252**, 1162–1164 (1991).

54. Marchler-Bauer, A. *et al*. CDD/SPARCLE: Functional classification of proteins via subfamily domain architectures. *Nucleic Acids Res*. **45**, D200–D203 (2017).
